# The fractal characteristics of pore size distribution in cement-based materials and its effect on gas permeability

**DOI:** 10.1038/s41598-019-53828-5

**Published:** 2019-11-20

**Authors:** Jie Zhu, Rui Zhang, Yang Zhang, Fa He

**Affiliations:** School of Mechanics & Civil Engineering, China University of Mining & Technology (Beijing), Beijing, 100083 China

**Keywords:** Civil engineering, Composites

## Abstract

To study the influence of the pore structure of cement-based materials on macroscopic features (gas permeability), mercury intrusion porosimetry (MIP) and nitrogen adsorption (NA) were applied to 8 groups of paste and mortar samples (including adding mineral admixtures or not and standard or sealed curing conditions). Pore size distribution has a great influence on gas permeability. By calculating pore surface fractal dimensions based on Zhang’s fractal model, the obvious fractal characteristics of micropores (<100 nm) and macropores (> 10^5^ nm) have been found. The pore diameter of the paste is mostly distributed in the micropores, and the average critical pore diameter is 82 nm. For mortar, the pore diameter is mostly distributed in the micropores and transition pores, and the average critical pore diameter is 121 nm, which means that the seepage pore diameters of the paste and mortar are 82 nm and 121 nm, respectively. The pore surface fractal dimensions of the visible pores are larger than those of the micropores, and there is an inverse relationship between the pore surface fractal dimensions and gas permeability. An important guide for engineering production is to use standard curing and add mineral admixtures to mortar materials to improve the impermeability as much as possible, while a contrary condition exists for paste materials.

## Introduction

Cement-based cementitious materials are the most widely and frequently used building materials in modern society, with a kind of non-uniform and multi-phase complex structural system whose non-linear mechanical characteristics of its macroscopic phenomenon reflect the complexity of its internal microstructure^1^. Pores and cracks with different sizes and contents (such as residual air bubbles, capillary pores, cementing pores, and fine cracks caused by the drying shrinkage of cementitious materials) in the cement-based materials developed after hardening are crucial components of their inner structure and are called pore structures. The pore structure characteristics, a significant subject of mesoscopic research in cement-based materials science, are closely related to the mechanical properties and durability of cement-based materials and seriously affect impermeability^2–6^.

Many factors are closely related to the pore structure of cement-based materials, such as the water-cement ratio^7–9^, curing conditions^10,11^ and mineral admixtures^12,13^, and mechanical effects in the process of preparation, curing and engineering applications also cause changes in the pore structure of materials and thus affect gas permeability. Sabine C showed that the pore size distribution of mortar was related to the mixing parameters, and the gas permeability depended on the mixture (water-cement ratio, aggregate volume content and aggregate particle size)^14^. Current measurement methods to determine pore size distribution include small-angle X-ray scattering (SAXS)^15,16^, atom force microscopy (AFM)^17,18^, micro-focus X-ray computed tomography (μCT)^19–21^ and scanning electron microscopy (SEM), categorized as physical methods^22,23^. Mercury intrusion porosimetry (MIP) and gas adsorption methods are adopted in terms of chemistry methods, and gas adsorption is mainly carbon dioxide adsorption (CA) and low-temperature liquid nitrogen adsorption (NA)^24–27^. Zhongwei W *et al*. found through the SEM method that the inner pore structure of cement-based materials was very complex and disordered. They proposed to observe the changes from macro to micro from the perspective of macroscopic properties and microscopic pores^28^.

Based on this idea, this paper studies cement paste and mortar materials to determine the correlation between the microscopic pore structure and macroscopic gas permeability. Currently, the mercury intrusion method is widely used in the study of pore structure and gas permeability. Kefei L *et al*. analysed the pore structure of cement-based materials under different curing conditions based on the mercury intrusion method and found that curing conditions had a significant impact on the pore size distribution^29^. By using the mercury intrusion method and nuclear magnetic resonance (NMR), Junzhi Z showed that there was a correlation between the gas permeability and porosity of cement-based materials^2^. By mercury intrusion and gas adsorption tests, Hamani showed that the porosity was not enough to predict the permeability regardless of mix proportion. It was necessary to take the microstructure pore size into account, and gas permeability appeared to be well correlated to the main pore diameter mode deduced from MIP^30^. Yuya’s research showed that the median pore diameter obtained by seepage theory has a high determination coefficient^31^. Heede studied the pore structure of concrete mixtures with different fly ash contents, and a 50% fly ash mixture by mass with a binder content of 400 kg/m3 and a water-to-binder ratio of 0.4 had a lower capillary gas permeability (78.9%) than a proper reference normally used in this environment^32^. However, the mercury intrusion method has some limitations in analysing pore structure, especially in measuring the actual pore size distributions^33,34^. In this paper, the data of mercury intrusion and nitrogen adsorption were combined to analyse the pore size distribution of cement-based materials more comprehensively.

To realize microscopic pore characteristics more concretely, it is necessary to develop a theory to quantitatively describe the pore structure of materials. Fractal geometry is a new branch of mathematics applied to describe the irregular and disordered phenomena and behaviours in nature, which can quantitatively describe the complexity of the geometric component. Because of the complex heterogeneity and self-similarity of pore structure^35,36^, we can apply fractal theory to establish the calculation model of pore structure and calculate the fractal dimension by the mathematical method for investigating the relationship between fractal dimension and the macroscopic mechanical properties and durability of cement-based materials. In recent years, some fractal models, such as the pore axis fractal model^37^,the space-filling model^38^, the pore cross-section contour model^39^, and the Menger sponge model^40^ have been applied to analysing the pore structures of cementitious materials. However, since these models were developed from different cementitious materials, they may have limited accuracy and reliability when applied to cement-based materials^41,42^. Zhang’s model^43^ is adopted in this study, which is the thermodynamic equilibrium between the increase of surface energy and the penetration work of mercury in the process of MIP measurement. It is considered suitable to apply this model for evaluating pore-size distributions of the specimens in MIP measurement^42^. Qiang Z *et al*. calculated the pore surface fractal dimensions of cement-based materials by using Zhang’s model and concluded that the pore surface fractal dimension can be considered an indicator to characterize the microstructure of multi-porous cement-based materials^10^. Jiyoung K, using Zhang’s model, calculated the surface fractal dimension of high strength cement paste in different pore diameter range by using the results of MIP, and the results showed that the ratio of the surface fractal dimension to the volume of larger capillary pores was strongly correlated with the compressive strength of the specimens^42^.However, the relationship between the fractal dimension and gas permeability can be further studied. In this paper, 8 groups of cement paste and mortar samples with different curing conditions and mineral admixtures were produced. A series of pore characteristics and gas permeability tests, including porosity, mercury intrusion porosimetry (MIP) and nitrogen adsorption (NA) testing were conducted. By calculating the pore surface fractal dimensions of cement paste and mortar samples, the relationship of the pore surface fractal dimensions and gas permeability is studied.

## Experimental Design and Methods

### Samples preparation

The cementitious materials include mineral admixtures and P.O42.5 cement. The initial setting time of P.O42.5 is 95 min, and the final setting time is 140 min. Mineral admixtures consist of fly ash, mineral powder and silica fume whose chemical compositions are shown in Table 1. The natural river sand with volume density (1.57 g/cm^3^) was used. Table 2 shows the proportions of the paste and mortar samples. The mixing operation was conducted in accordance with GB50080-2016 (Performance test method standard for ordinary concrete mixtures). Eight cylinders were made with a size of 25 mm × 50 mm and were placed on a shaking table to vibrate for 60 s until no obvious bubbles turned up. Finally, the samples were stored in the laboratory after being dried at normal temperature (25 ºC) for 1 d, and all the models were removed. Half of the cylinder samples were randomly selected and cured in the standard curing room (ambient temperature is 20 ±2 °C, and humidity is not less than 95%). The other half of the samples were loaded into sealing bags and placed in the curing room for sealed curing. After curing for 28 d, all samples were put in the laboratory with natural temperature and humidity. Data on all eight samples are shown in Table 3. Single-variable factors were controlled, for example, samples No. 2 and No. 3 (No. 6 and No. 7) can be used to compare the effects of curing conditions on cement-based materials, and samples No. 3 and No. 4 (No. 6 and No. 8) can be used to compare the effects of adding mineral admixtures.

**Table 1:** Chemical composition of cementitious materials.

cementitious materials	chemical component (%)						
	SiO_2_	Al_2_O_3_	Fe_2_O_3_	CaO	TiO_2_	MgO	Na_2_O
cement	21.58	4.03	3.46	61.49	—	2.60	0.51
fly-ash	56.74	24.59	6.55	4.87	1.87	—	—
mineral powder	21.45	9.68	33.7	23.52	1.12	6.26	—
silica fume	96.74	0.32	0.08	0.11	—	0.10	0.09

**Table 2:** Sample quality ratio.

Sample ID	Mix proportion (kg/m³)					
	cement	fly-ash	mineral powder	silica fume	water	sand
No. 1	1712	—	—	—	513	—
No. 2	1253	90	45	15	702	—
No. 3	1253	90	45	15	702	—
No. 4	1429	—	—	—	714	—
No. 5	575	—	—	—	173	1725
No. 6	526	37.8	18.9	6.3	295	1578
No. 7	526	37.8	18.9	6.3	295	1578
No. 8	528	—	—	—	264	1584

**Table 3:** Sample design.

Sample ID	material	w/b ratio	Add mineral	Curing condition	Specimen code
			admixture		
No. 1	paste	0.3	NO	control group curing	P-N-C
No. 2		0.5	YES	control group curing	P-Y-C
No. 3		0.5	YES	sealed curing	P-Y-S
No. 4		0.5	NO	sealed curing	P-N-S
No. 5	mortar	0.3	NO	sealed curing	M-N-S
No. 6		0.5	YES	control group curing	M-Y-C
No. 7		0.5	YES	sealed curing	M-Y-S
No. 8		0.5	NO	control group curing	M-N-C

P is paste, M is mortar. Y represents adding mineral admixture, N represents without mineral admixture. C is control group curing (standard curing), S is sealed curing.

### Experimental scheme and design

Eight cylinders were used to measure porosity and gas permeability, and then the fragments (approximately 1 cm on each side) of each sample were peeled for testing pore characteristics, including nitrogen adsorption (NA) and mercury intrusion porosimetry (MIP).

Porosity and gas permeability are vital parameters to describe the impermeability characteristics of cement-based materials. First, the samples were put into the CMS 300 Permeameter holder, the confining pressure of which was 500 psi. We loaded the eight cylinders into the rotary table at one time and input the geometric parameters of each sample into the software. Second, the test machine was powered on to preheat for 30 min. Before the porosity and gas permeability testing, the test programme was started to input the atmospheric pressure, experimental temperature data under the test environment, test number and the basic parameter data. There was a gas container filled with a certain pressure at the inlet. The dry air with 200Psi pressure was forced in the sample to the porosity of each sample can be calculated by using Boyle’s law. When the measurement started, the system automatically connected the container to the inlet and then measured the change in pressure over time. According to the change in pressure over time and related parameters, the computer automatically collected and stored data. The permeability of each sample can be calculated by using the integrated form of the combined Darcy, Klinkenberg and Forchheimer equations. When the test was over, the system automatically released the radial and axial pressure and applied a radial vacuum to return the sample to the rotary table. At the same time, the line printer automatically printed out the measurement results.

Mercury intrusion porosimetry (MIP) and nitrogen adsorption methods (NA) have commonly been used in recent years to evaluate the pore characteristics of cement-based materials. For observing nitrogen adsorption isotherms at a temperature of 77 K, the gas adsorption was experimented with the ASAP2020 automatic at the Comprehensive Thermal Physics Laboratory of Tsinghua University in China. As the relative pressure (P/P_0_) increases from 0 to 1, we can test the quantity of liquid nitrogen adsorbed. The testing pore diameter range is from 271.6 nm to 1.7 nm. Before testing, the samples were dried overnight to remove air and water at 105 °C in a vacuum oven. Then, mercury intrusion porosimetry (MIP) was conducted using an AutoPore IV 9500 Instrument. MIP is based on the functional relationship between the volume of intruded mercury and the intruded pressure to calculate the cumulative volume of the pores with different diameters. The sample size for the MIP experiments was approximately 1 cm × 1 cm. The cement-based material samples were dried at 60 °C for 12 hours and evacuated from the low-pressure port to <50 mm Hg to remove the residual gas and moisture. The Washburn equation was used to analyse the experimental data, and the pore diameter could be evaluated using the contact angle of 130° between the mercury and the pore surface. The surface tension is 485 dyn/cm2. The highest pressure in the mercury intrusion process can be more than 400 MPa. With increasing mercury pressure, the pores, the diameter of which varies from 360 μm to 3 nm, are gradually measured according to the simplified Washburn formula as follows:

1 $${\rm{d}}=\frac{-4{\rm{\gamma }}\,\cos \,{\rm{\theta }}}{{\rm{P}}}$$

where *P* is the mercury intrusion pressure (N/m^2^), *d* is the pore diameter (m), *γ* is the surface tension of mercury (N/m^2^) and θ is the contact angle between the mercury and the pore wall.

## Results and Analysis

### Porosity and gas permeability

In Table 4, comparing 8 groups of porosity and gas permeability values, it can be seen that there is a great difference in porosity between the paste and mortar, but not in gas permeability. In addition, the porosity values by MIP testing in Table 5 are not significantly different. This indicates that not all types of pores contribute to the gas permeability, and there might be some dominant pores that influence the gas permeability of the paste and mortar materials.

**Table 4:** Porosity and gas permeability test data.

Sample ID	Specimen code	Density (g/cm^3^)	Porosity (%)	Gas permeability (md)
No. 1	P-N-C	2.225	7.190	0.0147
No. 2	P-Y-C	2.105	8.345	0.2294
No. 3	P-Y-S	2.120	9.625	0.0920
No. 4	P-N-S	2.143	8.220	0.0807
No. 5	M-N-S	2.474	16.646	0.0163
No. 6	M-Y-C	2.461	13.012	0.0014
No. 7	M-Y-S	2.404	15.084	0.0026
No. 8	M-N-C	2.374	15.185	0.0707

**Table 5:** MIP test data.

Sample ID	Specimen code	MIP	Median	Average	Critical
		porosity (%)	pore	pore	pore
			diameter (nm)	diameter (nm)	diameter (nm)
No. 1	P-N-C	16.386	36.6	19.0	53.8
No. 2	P-Y-C	22.092	37.0	16.1	100.2
No. 3	P-Y-S	19.815	40.5	16.6	95.3
No. 4	P-N-S	21.261	77.2	28.3	77.2
No. 5	M-N-S	18.251	334.9	55.4	120.7
No. 6	M-Y-C	18.990	65.3	23.4	120.9
No. 7	M-Y-S	18.870	97.3	27.1	120.8
No. 8	M-N-C	17.663	61.5	25.4	120.7

For the paste and mortar samples, the effect of curing condition on the gas permeability is reversed. The gas permeability of the paste samples under sealed curing is less than that of the samples under standard curing, as shown by comparing sample No. 2 (P-Y-C) with No. 3 (P-Y-S). However, the gas permeability of the mortar samples increased under sealed curing, as shown by comparing sample No. 6 (M-Y-C) with No. 7 (M-Y-S). The effect of mineral admixtures on gas permeability for the paste and mortar was also different. By adding mineral admixtures, the gas permeability of the paste increased, as shown by comparing sample No. 3 (P-Y-S) with No. 4 (P-N-S). However, comparing sample No. 6 (M-Y-C) with No. 8 (M-N-C), the gas permeability of the mortar decreases. This decrease indicates that for two different cement-based materials, the paste and the mortar, different curing conditions are required to configure favourable impermeability. There is no need to add mineral admixtures to paste material (This is also consistent with the actual engineering production of paste material), and the curing environment requires sealing, meaning that not too much water is used to participate during the curing period. For mortar, mineral admixtures are required to refine the particle gradation to fill larger pores, and it is better to adopt standard-condition curing to provide sufficient water for hydration reactions and produce finer gelation pores.

### MIP and NA results

As for porous materials, American Society for Testing and Materials (ASTM) stipulated that pores with diameters from 1.5 nm to 100 nm can be characterized by nitrogen adsorption^44^. In the soil and rock fields, ASTM recommends the mercury intrusion method to measure the pores from 2.5 nm to 10^5^ nm^45^. MIP and NA data can be used to analyse pore size distributions and types. Table 5 shows that the average median pores of the paste and mortar are 47.8 nm and 139.8 nm, respectively. The average pore diameter of the paste lies in the interval of 16.1 nm to 28.3 nm and 23.4 nm to 55.4 nm for the mortar, which demonstrates that the pore diameter distributions of the paste and mortar are quite different.

**Figure 1:**
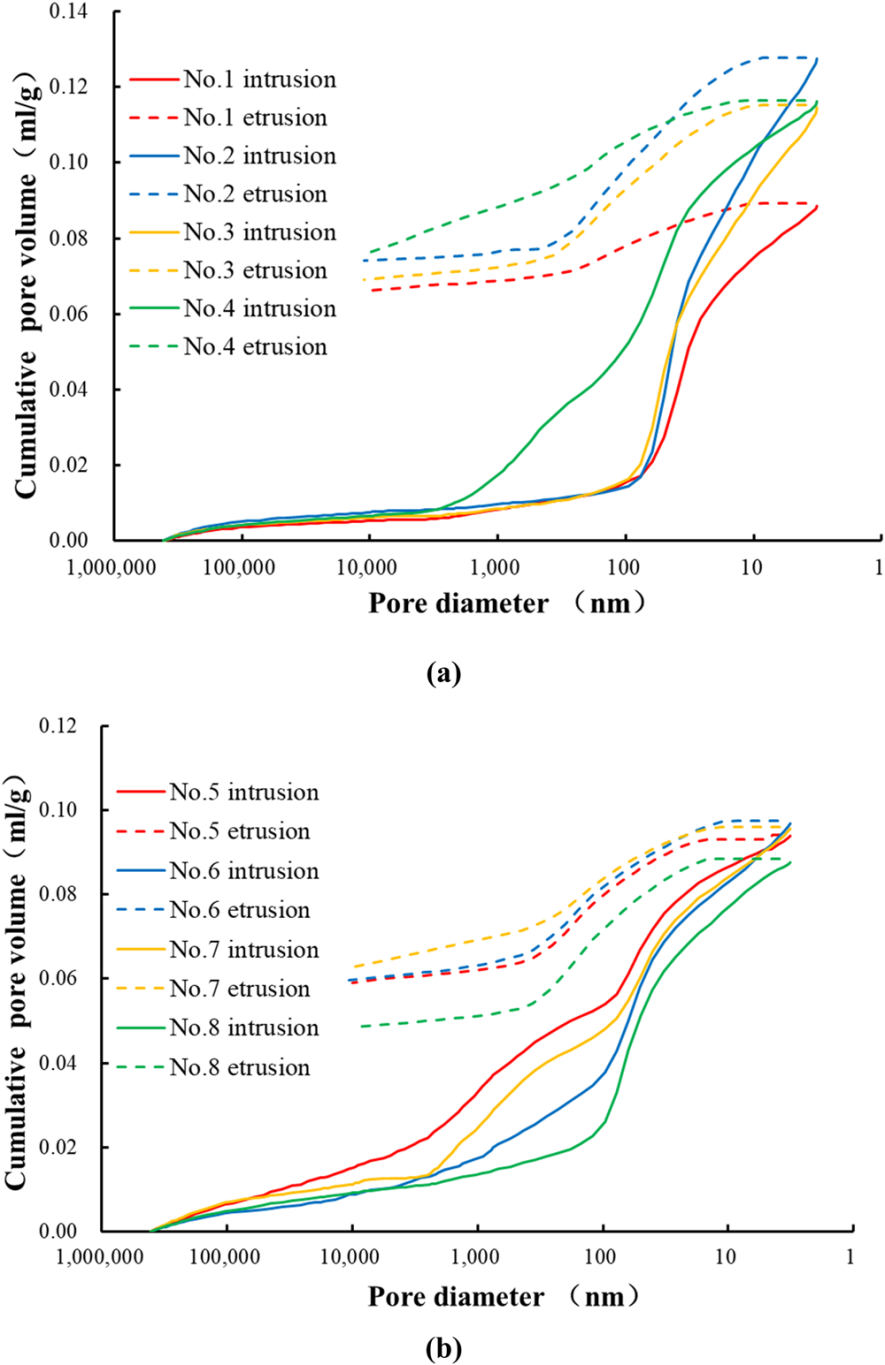
Mercury intrusion porosimetry curves of (**a**) cement paste and (**b**) cement mortar.

The pore network in cement-based materials is interconnected with randomly distributed systems. Metha proposed the concept of critical pore size, which is the corresponding pore size when the cumulative mercury volume curve begins to increase substantially, as a significant parameter characterizing the relationship between the gas permeability and pore size distribution^46^. The critical pore diameter is the maximum pore diameter that can connect the larger pores and reflects the connectivity of the pores. The pores larger than the critical pore diameter affect gas permeability. The critical pore diameter can be concluded from the MIP curve in Fig. 1. As shown in Table 5, the critical pore diameter of the paste is located in the interval of 53.8 nm to 100.2 nm, with an average value of 82 nm. The critical pore diameter of the mortar is approximately 120 nm, with an average value of 121 nm.

**Figure 2:**
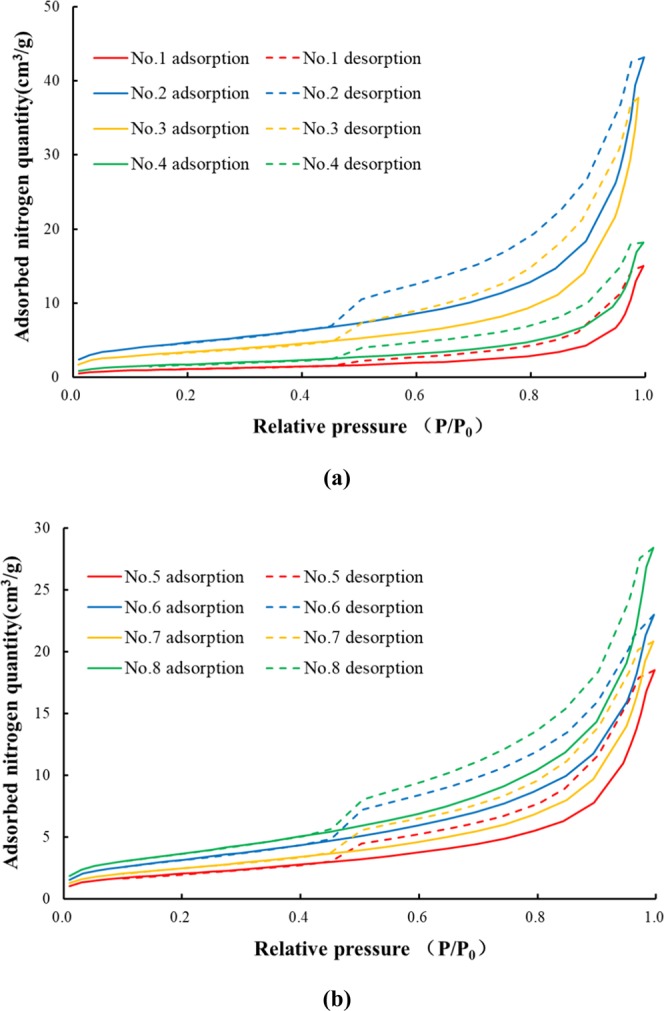
Nitrogen adsorption and desorption curves of (**a**) cement paste and (**b**) cement mortar.

Figures 1 and 2 are mercury intrusion porosimetry curves and nitrogen adsorption and desorption curves, respectively, of the paste and mortar, which can be used to analyse the pore characteristics of cement-based materials. In Fig. 1, the area enclosed by the mercury intrusion porosimetry curve is called the hysteretic loop, which is formed by the open pores existing in the cement-based materials. These open pores represent mercury left in the process of mercury extrusion. It can be seen that the hysteretic loop of the paste is wider than that of the mortar, meaning that the open pore volume of the paste is obviously larger than that of the mortar. The average porosity of the paste is half of the average porosity of the mortar, but the average gas permeability is 5 times that of the mortar, indicating that open pores are dominant in influencing the gas permeability of cement-based materials. In addition to the open pores, the pores in cement-based materials consist of semi-open pores and closed pores. The closed pores cannot be measured in MIP, and the mercury in the semi-open pores can be completely extruded, indicating that the total amount of mercury intruded minus the volume of the open pores is the volume of the semi-open pores. In Fig. 2, the adsorption isotherm shows an obvious upward trend within the range of the relative pressure, which is greater than 0.8. Except for sample No. 1, when the relative isotherm pressure of all the samples is approximately 0.5, the adsorption isotherm curve drops sharply, which indicates ink-bottle pores.

### MIP data corrected

Cement-based materials are compressible, and a matrix compression phenomenon exists in the mercury intrusion phase^47,48^. Due to the limitation of the experimental conditions, high pressure may compress cement-based material matrix and damage fine pores, resulting in the measured pore volume being more than the real pore volume. In previous studies, the error caused by cementitious matrix compression was not taken into consideration when using MIP data to study pore size distribution. Therefore, we should correct the mercury intrusion data with nitrogen data to obtain the real pore volume of cement-based materials.

The compressibility *K*_*c*_ (cm^2^/N) of the cement-based material matrix is defined as^49^:

2 $${{\rm{K}}}_{{\rm{c}}}=\frac{{{\rm{dV}}}_{{\rm{c}}}}{{{\rm{V}}}_{{\rm{c}}}{\rm{dp}}}$$

where *V*_*c*_ (cm^3^/g) is the cement-based material matrix volume and $\frac{d{V}_{c}}{dp}$ represents the cement-based matrix volume change as a function of the pressure.

For a compressible solid^49^:

3 $${\Delta V}_{{\rm{obs}}}={\Delta V}_{{\rm{p}}}+{\Delta V}_{{\rm{c}}}$$

where *ΔV*_*obs*_ (cm^3^/g)*, ΔV*_*p*_ (cm^3^/g) and *ΔV*_*c*_ (cm^3^/g) are the changes in the observed mercury volume, pore-filling volume, and cement-based matrix compression volume, respectively.

**Figure 3:**
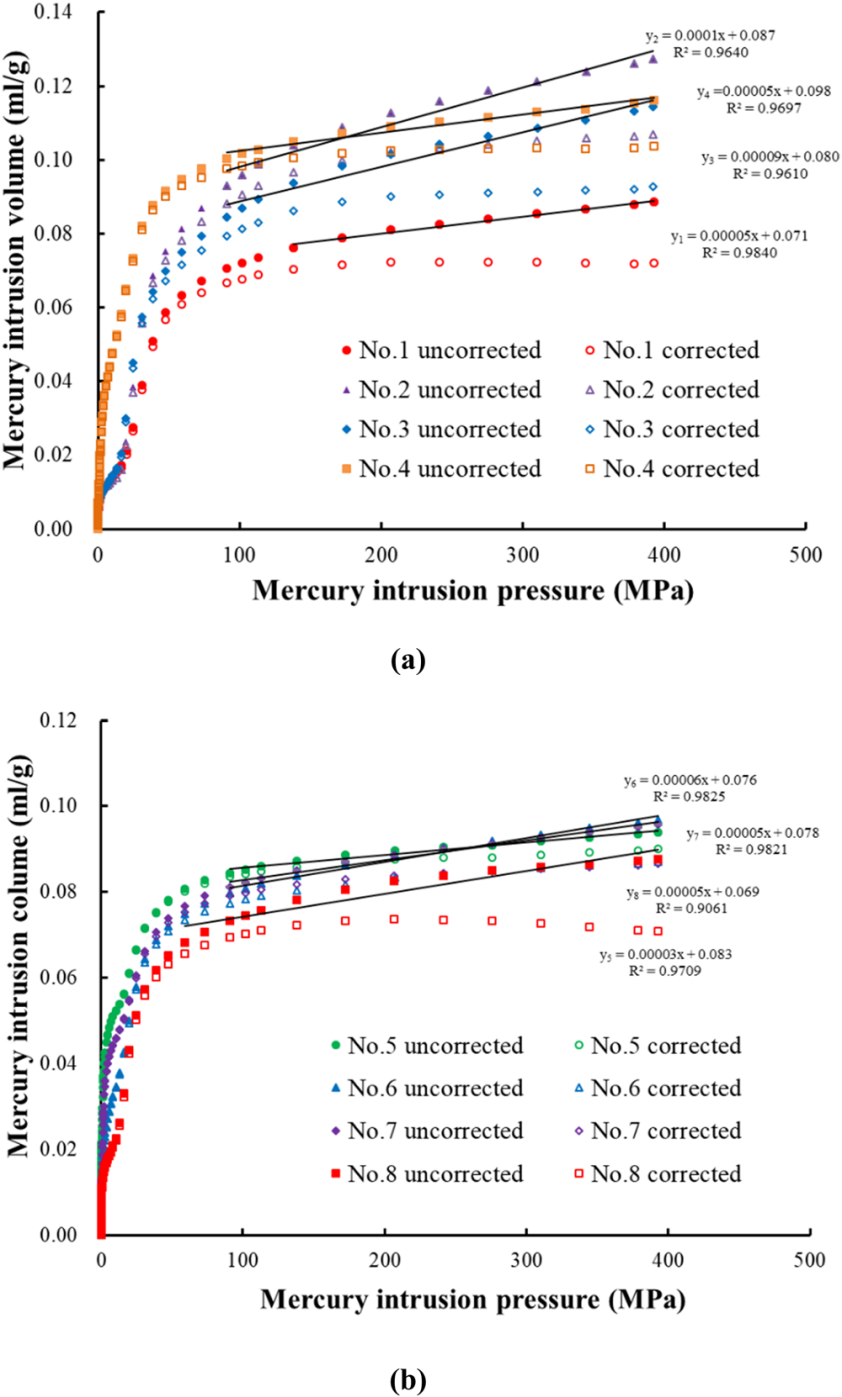
The cumulative mercury intrusion volumes before and after correction for (**a**) cement paste and (**b**) cement mortar.

As shown in Fig. 3, when the pressure is greater than 100 MPa, there is a roughly linear relationship between the mercury intrusion volume and the mercury intrusion pressure. Therefore, it can be considered that ΔV_obs_/ΔP is a constant defined as *β* when the mercury pressure exceeds 100 MPa. The constant ΔV_c_/ΔP can be defined as^49^:

4 $$\frac{{\Delta V}_{{\rm{c}}}}{\Delta P}={\rm{\beta }}-\frac{{\sum }_{{{\rm{R}}}_{{\rm{\min }}}}^{{{\rm{R}}}_{{\rm{\max }}}}{\Delta V}_{{\rm{p}}}}{\Delta P}$$

where *Rmax* (nm) and *Rmin* (nm) are the maximum and minimum pore diameters, respectively, corresponding to the pore volumes that need to be corrected. The real matrix volume *V*_*c*_ of cement-based materials can be obtained from the real density and sample weight. Therefore, it is necessary to correct the real pore volume by combining the MIP and NA data. The cumulative mercury intrusion volume before and after correction is shown in Fig. 3. Substituting Eq. (2) into Eq. (4), we can obtain Eq. (5) and calculate the compressibility *K*_*c*_ (cm^2^/N) of the coal matrix, whose results are shown in Table 6.

5 $${{\rm{K}}}_{{\rm{c}}}=({\rm{\beta }}-\frac{{\sum }_{{{\rm{R}}}_{{\rm{\min }}}}^{{{\rm{R}}}_{{\rm{\max }}}}{\Delta V}_{{\rm{P}}}}{\Delta P}){/V}_{{\rm{c}}}$$

**Table 6:** Correction of MIP test data.

Sample ID	Specimen	Kc × 10^−6^	β	R2	Cumulative pore	Corrected cumulative
	code	(cm^2^/N)	(10^−5^)		volume (ml/g)	pore volume (ml/g)
No. 1	P-N-C	50	6.84	0.9840	0.0886	0.0720
No. 2	P-Y-C	100	11.82	0.9604	0.1274	0.1070
No. 3	P-Y-S	90	10.58	0.9614	0.1145	0.0927
No. 4	P-N-S	50	7.32	0.9697	0.1162	0.1038
No. 5	M-N-S	40	5.59	0.9657	0.0837	0.0735
No. 6	M-Y-C	60	7.19	0.9825	0.0968	0.0870
No. 7	M-Y-S	50	6.45	0.9821	0.0957	0.0866
No. 8	M-N-C	50	5.73	0.9061	0.0876	0.0709

In Fig. 3, by comparing the paste with the mortar under the same curing conditions and mineral admixtures, it can be seen that the paste shows an obvious difference in pore volume before and after correction, while there is no significant difference in the pore volume of the mortar. In Table 6, the average compressibility of the paste material is 7.25 × 10^−5^, and the average compressibility of the mortar material is 4.75 × 10^−5^, which means that the greater the content of cementitious materials is, the greater the influence on the matrix compression. Through the comparison of the pore volumes before and after correction, the matrix compressibility of the cement-based materials cannot be ignored. When describing the pore characteristics of cement-based materials by MIP, it is necessary to use nitrogen adsorption data to correct the pore volume to reduce the influence of matrix compression on the process of mercury intrusion.

## Discussion

### The relationship between gas permeability and pore surface fractal dimensions

The fractal dimension is a geometric representation of the pore surface and boundary, reflecting the availability of space occupied by complex structures. It is a measure of the irregularity of complex structures^46^. In Table 4, there is no distinct relevance of the gas permeability and porosity of the testing samples; thus, fractal theory is applied to study the relationship between the pore size distribution and gas permeability. The pore surface fractal dimension is an indicator of the microstructure of cement-based multi-porous materials, which reflects the roughness of the porous material surface^10^. Zhang *et al*. established the pore surface fractal dimension formula of internal pore structure based on the equilibrium relationship between the increased surface energy and the mercury intrusion volume in MIP^43^.

6 $$\mathrm{ln}(\frac{{{\rm{W}}}_{{\rm{n}}}}{{{\rm{r}}}_{{\rm{n}}}^{2}})={{\rm{D}}}_{{\rm{s}}}\,\mathrm{ln}(\frac{\sqrt[3]{{{\rm{V}}}_{{\rm{n}}}}}{{{\rm{r}}}_{{\rm{n}}}})+{\rm{C}}$$

where *W*_*n*_ is the cumulative intrusion work, *V*_*n*_ (ml/g) is the mercury intrusion volume, *r*_*n*_ (nm) is the pore diameter that can be tested in the first step of *n*, and *C* is the model constant.

7 $${{\rm{W}}}_{{\rm{n}}}={\sum }_{{\rm{i}}=1}^{{\rm{n}}}{{\rm{P}}}_{{\rm{i}}}{\Delta V}_{{\rm{i}}}$$

where *P*_*i*_ and *V*_*i*_ are the mercury intrusion pressure and the mercury volume, respectively, at each step. The pore surface fractal dimension *D*_*s*_ value is the slope of the curve with $\mathrm{ln}(\frac{\sqrt[3]{{{\rm{V}}}_{{\rm{n}}}}}{{{\rm{r}}}_{{\rm{n}}}})$ as the abscissa and $\mathrm{ln}(\frac{{{\rm{W}}}_{{\rm{n}}}}{{{\rm{r}}}_{{\rm{n}}}^{2}})$ as the ordinate.

**Figure 4:**
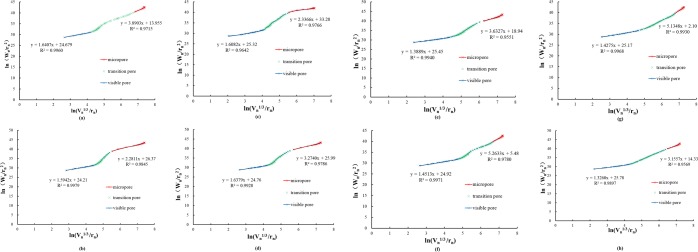
Pore surface fractal of the micropore and visible pore regions for samples (**a**) No. 1 (P-N-C), (**b**) No. 2 (P-Y-C), (**c**) No. 3 (P-Y-S), (**d**) No. 4 (P-N-S), (**e**) No. 5 (M-N-S), (**f**) No. 6 (M-Y-C), (**g**) No. 7 (M-Y-S) and (**h**) No. 8 (M-N-C).

As can be clearly observed in Fig. 4, all of the pore surface fractal dimension curves were divided into three specific regions. The pore surface fractal characteristics of cement-based materials are more obvious in pores with diameters less than 100 nm and more than 10^5^ nm. Therefore, according to the pore surface fractal phenomenon, the pore size can be divided into the micropore region (<100 nm), the transition pore region (100–10^5^ nm) and the visible pore region (>10^5^ nm). The micropore region was dominated by cementitious pores and capillary pores, while the visible pore region was dominated by visible pores and cracks. The slopes of the microporous region and the visible pore region are the pore surface fractal dimensions. In Table 7, there is little difference in the pore surface fractal dimension *D*_*s1*_ between the paste and mortar in the visible pore region, with average values of 1.62 and 1.40, respectively. However, the *D*_*s2*_ of the paste and mortar samples are apparently different in the micropore region, with average values of 2.95 and 4.30, respectively. There are significant differences in the gas permeability between the two materials, which indicates that the microporous region is the key to determining the gas permeability of cement-based materials. The pore surface fractal dimension is an indicator of the roughness of the pore surface, indicating that the larger *D*_*s2*_ is, in the micropore region, the more complex the pore structure surface. The pore surface of the mortar samples is more complex than that of the paste samples.

**Table 7:** Pore surface fractal dimension.

	Paste				Mortar			
	No.1	No.4	No.3	No.2	No.6	No.7	No.5	No.8
Visible pore region *D*_*s1*_	1.64	1.64	1.61	1.59	1.45	1.43	1.39	1.33
Micropore region *D*_*s2*_	3.89	3.27	2.34	2.28	5.26	5.13	3.63	3.16
Permeability (md)	0.0147	0.0807	0.0920	0.2294	0.0014	0.0026	0.0163	0.0707

In Table 7, the pore surface fractal dimensions *D*_*s1*_ and *D*_*s2*_ are numerically smaller with increasing gas permeability, meaning a negative increasing trend. A larger pore surface fractal dimension means more micropore content from the perspective of the pore surface fractal dimension numerical analysis of material gas permeability, meaning lower seepage pore content and gas permeability, which increases the impermeability performance and improves the durability of the cement-based material.

### The relationship between gas permeability and pore size distribution

**Figure 5:**
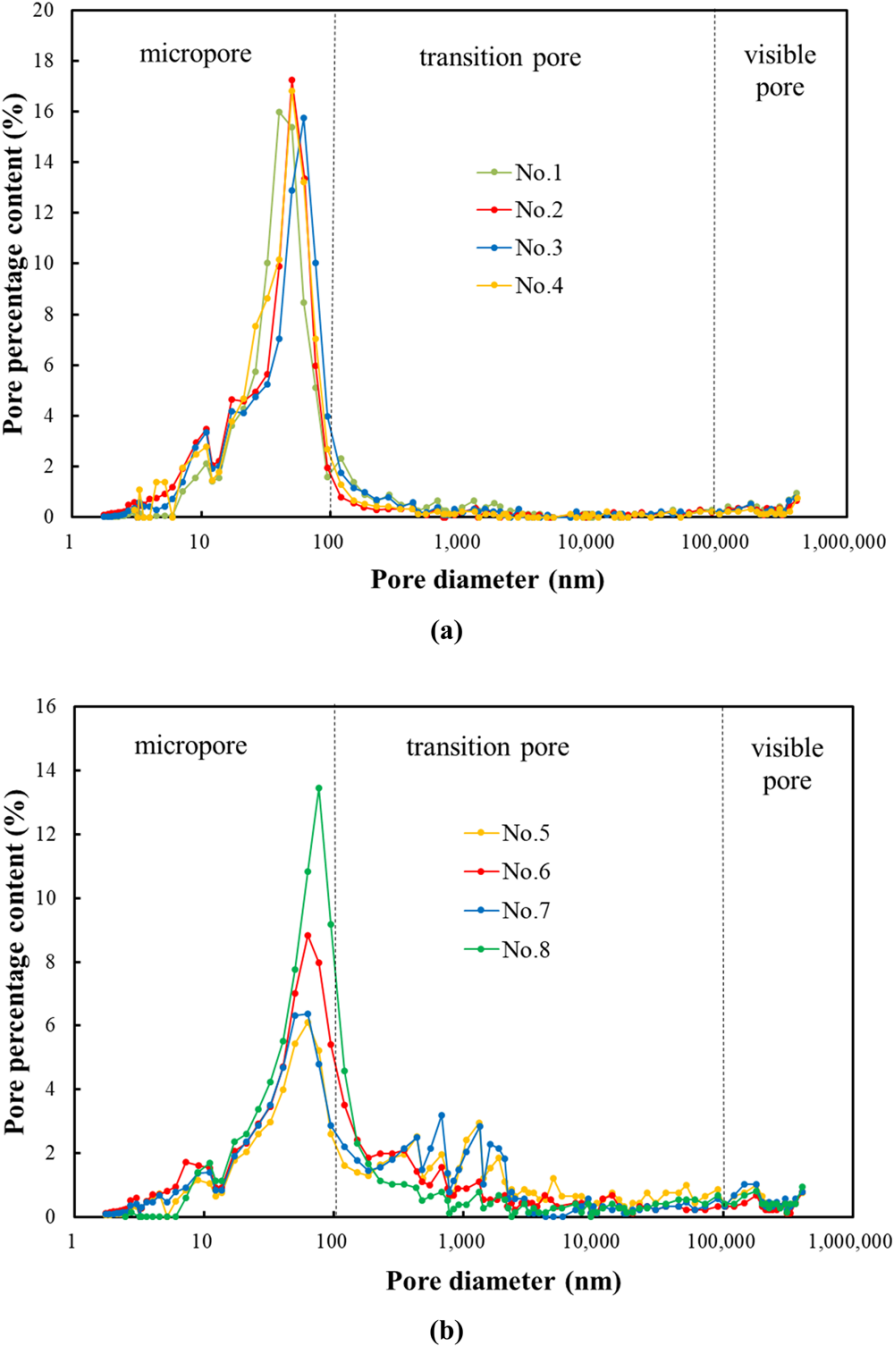
Pore size distributions of the (**a**) cement paste and (**b**) cement mortar.

**Figure 6:**
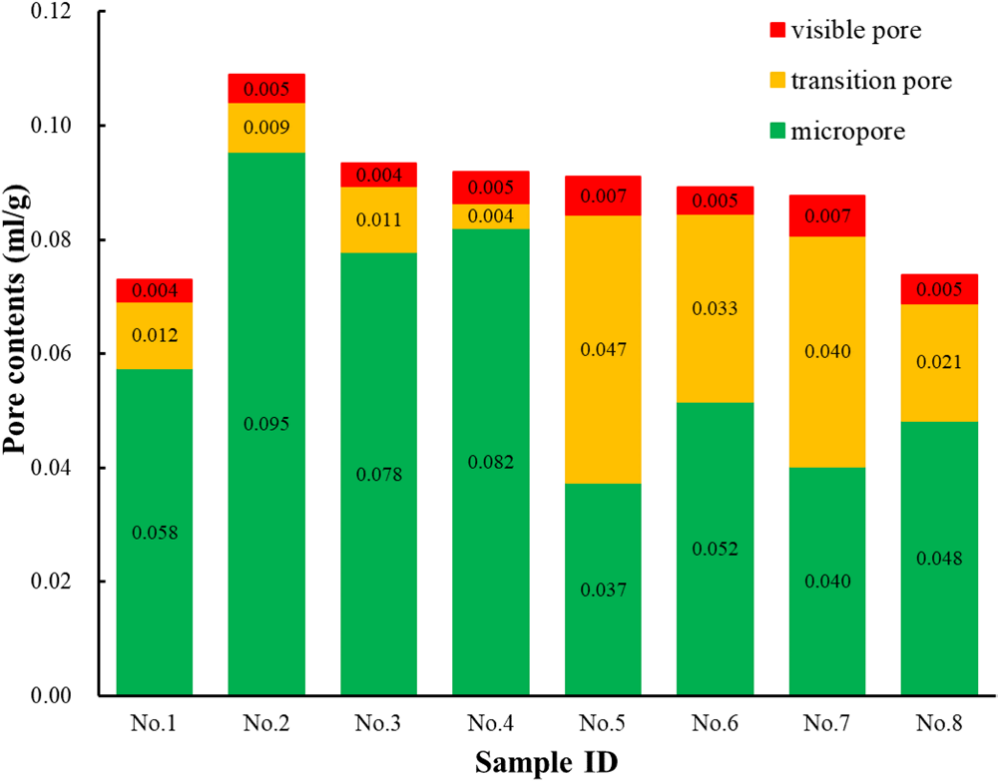
Pore size distributions of the paste and mortar.

In this experiment, there is no significant correlation between the permeability and porosity, which further indicates that it is essential to study the pore size distribution of cement-based materials and its effect on gas permeability. In Fig. 5, it can be seen from the corrected pore size distribution that the maximum peak values of the paste and mortar are both in the micropore region, indicating that the pore size distribution of the cement-based materials is mainly composed of micropores. The mortar shows the secondary peak in the transition pore region, which means that the mortar pore size distribution in the transition area is relatively complex. As can be clearly seen in Fig. 6, the average content of the micropores, transition pores and visible pores in the paste (No. 1, No. 2, No. 3 and No. 4) are 0.075 cm^3^/g, 0.009 cm^3^/g, and 0.0045 cm^3^/g, respectively, which accounts for 85%, 10% and 5%, indicating that the pore size distribution of the paste is mainly below 100 nm and the critical pore diameter of the paste is 82 nm in Table 5. Therefore, it can be concluded that the sizes of the seepage pores in the paste are mainly distributed between 82 and 100 nm. However, the average contents of the micropores, transition pores and visible pores of the mortar (No. 5, No. 6, No. 7 and No. 8) are 0.033 cm^3^/g, 0.035 cm^3^/g, and 0.006 cm^3^/g, which account for 51%, 41% and 8%, respectively, and the average content of the transition pores of the mortar is approximately 4 times that of the paste. The diameter of sand particles is larger than that of cement particles. The paste belongs to a single system of cement particles, while the mortar is a system that is composed of cement and sand. From the perspective of physical stacking, the pore sizes of particles with different diameters are larger, so the porosity of the mortar is larger than that of the paste. It can also be seen in Fig. 5 that the transition pores of the mortar are larger than those of the paste.

In Table 5, the critical pore diameter of the mortar is 121 nm, indicating that the pore size distributions that influence seepage in the mortar are transition pores with diameters greater than 121 nm and the visible pore region, but the content of the two regions is less than 50% of the total porosity, which can explain the confusing phenomenon of samples No. 6 and No. 7 with high porosity but low gas permeability. By comparing the paste samples No. 2 (P-Y-C) with No. 3 (P-Y-S) (or the mortar samples No. 6 (M-Y-C) with No. 7 (M-Y-S)), it can be seen that the peak content of micropores under sealed curing is less than that under standard curing. By comparing the paste samples No. 3 (P-Y-S) with No. 4 (P-N-S) (or the mortar samples No. 6 (M-Y-C) with No. 8 (M-N-C)), there is no significant change in the peak value of the micropores after adding mineral admixtures in the paste. However, as for the mortar material, the peak value of the micropores decreases obviously after adding mineral admixtures, meaning that the content of transition pores in the mortar samples increases with mineral admixtures.

## Conclusions

1. Different preparations of the cement-based samples lead to different pore characteristics, porosity and gas permeability. To obtain cement-based materials with high impermeability, the cement paste needs to be sealed without adding mineral admixture. For cement mortar, it is necessary to add mineral admixture to refine the grain gradation and adopt standard curing to promote hydration reactions to produce finer cementitious pores.
2. The micropore region (<100 nm) and visible pore region (>10^5^ nm) in the cement-based samples have obvious fractal characteristics, and the pore surface fractal dimension was calculated as a quantitative parameter to characterize the surface roughness of the pores. Micropores are the most closely related to the gas permeability of the cement-based materials. The lower the pore surface fractal dimension is, the better the gas permeability of the paste and mortar samples.
3. The gas permeability is mainly affected by the pore size distribution rather than the porosity. This shows that the pore size distributions of the paste and the mortar samples are different. There are many micropores in the paste samples, and the average critical pore diameter is 82 nm. For the mortar samples, most of the pores are micropores and transition pores, and the average critical pore diameter is 121 nm, indicating that the seepage pore diameters of the paste and mortar samples are larger than 82 nm and 121 nm, respectively.
